# Colonic Stenting as a Bridge to Surgery Versus Emergency Resection in Obstructive Colon Cancer: A Systematic Review of Surgical Outcomes

**DOI:** 10.3390/jcm15124416

**Published:** 2026-06-07

**Authors:** Adrian Marius Silaghi, Catalin Cicerone Grigorescu, Dragos Serban, Laura Carina Tribus, Vlad Denis Constantin, Ion Motofei, Dan Dumitrescu, Corneliu Tudor, Victor Dumitrescu, Bogdan Mihai Cristea, Tudor Mihai Badescu

**Affiliations:** 1 Doctoral School, “Carol Davila” University of Medicine and Pharmacy Bucharest, 020021 Bucharest, Romania; adrian-marius.silaghi@drd.umfcd.ro; 2 Department of General Surgery, St. Pantelimon Emergency Clinical Hospital, 021659 Bucharest, Romania; 3 Faculty of Medicine, “Carol Davila” University of Medicine and Pharmacy Bucharest, 020021 Bucharest, Romania; dan.dumitrescu@umfcd.ro (D.D.);; 4 Faculty of Medicine, “Vasile Goldis” Western University of Arad, 310025 Arad, Romania; 5 4th Department of General Surgery, Emergency University Hospital Bucharest, 050098 Bucharest, Romania; 6 Faculty of Dental Medicine, “Carol Davila” University of Medicine and Pharmacy Bucharest, 020021 Bucharest, Romania; 7 Faculty of Medicine, “Lucian Blaga” University of Sibiu, 550169 Sibiu, Romania

**Keywords:** colon cancer, bowel obstruction, emergency surgery, self-expanding metal stent (SEMS), staged surgery, systematic review, colorectal cancer surgery, bridge-to-surgery (BTS) strategies

## Abstract

**Background**: Acute malignant colorectal obstruction requires urgent management, traditionally by emergency resection or stoma formation. Colonic stenting as a bridge to surgery (BTS) has emerged as an alternative, but concerns persist regarding oncologic safety. This systematic review evaluates short-term and long-term outcomes of BTS with self-expanding metal stents (BTS-stent) compared with acute resection (AR). **Methods**: A systematic review was conducted on multiple databases. PubMed, Cochrane Library, and Google Scholar were searched for studies published between 2015 and 2025 reporting surgical management of obstructive colon cancer. Outcomes included perioperative morbidity and mortality, laparoscopic conversion, stoma formation, stent-related complications, and long-term overall survival (OS) and disease-free survival (DFS). Data were synthesized descriptively with reference to reported comparative effects and prior meta-analyses. **Results**: Fifteen studies published between 2015 and 2025 were included, encompassing 6999 patients (AR: 4944; BTS-stent: 1739; other BTS: 311). BTS was associated with higher rates of laparoscopic surgery (57% vs. 14%) and primary anastomosis, and significantly reduced stoma formation (27% vs. 60%). Postoperative morbidity and 30-day mortality were lower or comparable in BTS cohorts (4.9% vs. 7.8%). SEMS technical success ranged from 78 to 97%, with perforation rates of 0–15%, representing the main adverse event. Long-term outcomes were comparable between groups. Five-year OS ranged from 46 to 75% (BTS) and 46 to 74% (AR), with similar DFS. Subgroup data suggested possible worse outcomes in T4 tumors and improved results in selected stage III patients. Delayed surgery (>4 weeks) may improve survival, but increases complication risk. **Conclusions**: BTS with SEMS improves short-term outcomes without compromising long-term survival. Careful selection and avoidance of perforation are essential.

## 1. Introduction

Colon cancer is one of the most common malignancies worldwide, predominantly affecting individuals over the age of 50, with incidence continuing to rise over recent decades [1,2,3]. Despite advances in screening programs and improvements in early detection, a considerable proportion of patients still present with advanced disease, often complicated by acute surgical emergencies [4,5,6].

Malignant large bowel obstruction is a frequent and clinically significant complication of colon cancer, occurring in approximately 20–30% of cases [7,8]. It represents a life-threatening condition associated with severe physiological derangement, including fluid and electrolyte imbalance, systemic inflammatory response, and impaired nutritional status. These factors contribute to increased perioperative morbidity and mortality, reduced rates of primary anastomosis, and higher likelihood of permanent stoma formation [9]. Importantly, obstructive presentation is also considered a marker of advanced and biologically aggressive disease, which may negatively impact both short- and long-term outcomes.

The management of obstructive colon cancer remains challenging and is influenced by tumor location, disease stage, and the clinical condition of the patient [10]. Emergency colectomy with stoma formation remains a commonly performed treatment strategy; however, it is associated with substantial perioperative risk and a high rate of permanent stoma creation [11]. Alternatively, diverting stoma formation or bypass procedures may be used as temporizing measures to stabilize patients before definitive resection. Nevertheless, these approaches often require multiple procedures and are associated with prolonged treatment pathways and increased healthcare burden [12,13].

Self-expanding metallic stents (SEMSs) have emerged as an alternative bridge-to-surgery (BTS) strategy for malignant colonic obstruction, particularly in left-sided disease. SEMS placement allows rapid decompression of the obstructed colon, physiological stabilization, and conversion of an emergency surgical situation into an elective setting. This strategy has been associated with higher rates of minimally invasive surgery, increased primary anastomosis rates, and reduced need for permanent stoma formation [14,15,16,17]. However, concerns remain regarding oncologic safety, including the risk of microperforation, tumor dissemination, and potential adverse effects on long-term survival, with current evidence remaining controversial [18,19].

Although several systematic reviews and meta-analyses have evaluated SEMS in malignant colonic obstruction, important limitations persist. Many previous studies have combined heterogeneous patient populations, included both palliative and bridge-to-surgery indications, or relied on outdated evidence. In addition, differences in study design (randomized controlled trials versus observational studies) were not consistently addressed, and more recent high-quality studies have not been fully incorporated into pooled analyses. As a result, uncertainty remains regarding the comparative safety and effectiveness of SEMS versus emergency resection in contemporary clinical practice.

Therefore, the aim of this systematic review and meta-analysis was to evaluate the role of SEMS as a bridge-to-surgery strategy compared with emergency resection in patients with obstructive colon cancer, with a focus on short-term postoperative outcomes and long-term oncologic results. By incorporating the most recent evidence and critically appraising study-level limitations, this review seeks to provide an updated and clinically relevant synthesis to support decision-making in the management of malignant colonic obstruction.

## 2. Materials and Methods

### 2.1. Research Strategy

A systematic review was conducted in accordance with the Preferred Reporting Items for Systematic Reviews and Meta-Analyses (PRISMA) 2020 guidelines. 

A comprehensive literature search was performed in PubMed and the Cochrane Library to identify studies addressing the surgical management of colon cancer complicated by bowel obstruction or perforation. In addition, Google Scholar was used as a supplementary search tool to identify potentially relevant studies not captured by database searches. The search strategy combined controlled vocabulary and free-text terms related to colon cancer, acute complications, and emergency surgical management.

#### Literature Search Strategy

A comprehensive literature search was performed in PubMed/MEDLINE and the Cochrane Library to identify studies evaluating the surgical management of colon cancer complicated by bowel obstruction or perforation. In addition, Google Scholar was used as a supplementary source to identify potentially relevant studies not captured by database searches.

The literature search was conducted on 10 February 2026 and 10 March 2026, with the final search update performed on 25 March 2026.

The search strategy combined Medical Subject Heading (MeSH) terms and free-text keywords related to colon cancer and acute surgical complications. The following core search terms were used: “colon cancer,” “colorectal cancer,” “malignant bowel obstruction,” “intestinal obstruction,” “colonic obstruction,” “emergency resection,” “self-expanding metal stent,” and “bridge to surgery.” Boolean operators (AND/OR) were used to combine search concepts.

PubMed/MEDLINE search strategy:

(“colon cancer” OR “colorectal cancer”) AND

(“bowel obstruction” OR “intestinal obstruction” OR “malignant obstruction”) AND

(“emergency resection” OR “emergency surgery” OR “colectomy” OR “surgical management”) AND

(“self-expanding metal stent” OR SEMS OR stenting OR “bridge to surgery”)

Cochrane Library search strategy:

(“colorectal cancer” OR “colon cancer”) AND

(“bowel obstruction” OR “intestinal obstruction”) AND

(“stent” OR “self-expanding metal stent”) AND

(“emergency surgery” OR “resection”)

Only English language papers were included in the review. The search was limited to studies published between 2015 and 2025 to ensure inclusion of contemporary surgical practice.

### 2.2. Eligibility Criteria

Study eligibility was defined according to the PICO (Population, Intervention, Comparison, Outcomes) framework (Table 1).

Exclusion criteria were case reports, conference abstracts, duplicate publications, editorials, expert opinions, reviews, or meta-analyses, and studies addressing acute complications of benign disease.

### 2.3. Study Selection

Study selection was conducted in accordance with PRISMA 2020 guidelines. All records identified through database searching were imported into Zotero 9 reference management software for screening and duplicate removal. Two reviewers (A.M.S. and D.S.) independently performed the study selection in a two-stage process. In the first stage, titles and abstracts were screened to identify potentially relevant studies. Studies that clearly did not meet the inclusion criteria were excluded at this stage. In the second stage, the full texts of all potentially eligible articles were retrieved and independently assessed for inclusions according to the predefined eligibility criteria based on the PICO framework.

Each reviewer made independent decisions regarding inclusion or exclusion. Discrepancies between the two reviewers at either the title/abstract or full-text stage were resolved through discussion and consensus. If consensus could not be reached, a third senior reviewer (Author C) was consulted to make the final decision.

The reasons for exclusion of full-text articles were recorded and are presented in the PRISMA flow diagram. The entire screening process was performed independently by the reviewers to minimize selection bias and ensure methodological rigor.

**Figure 1:**
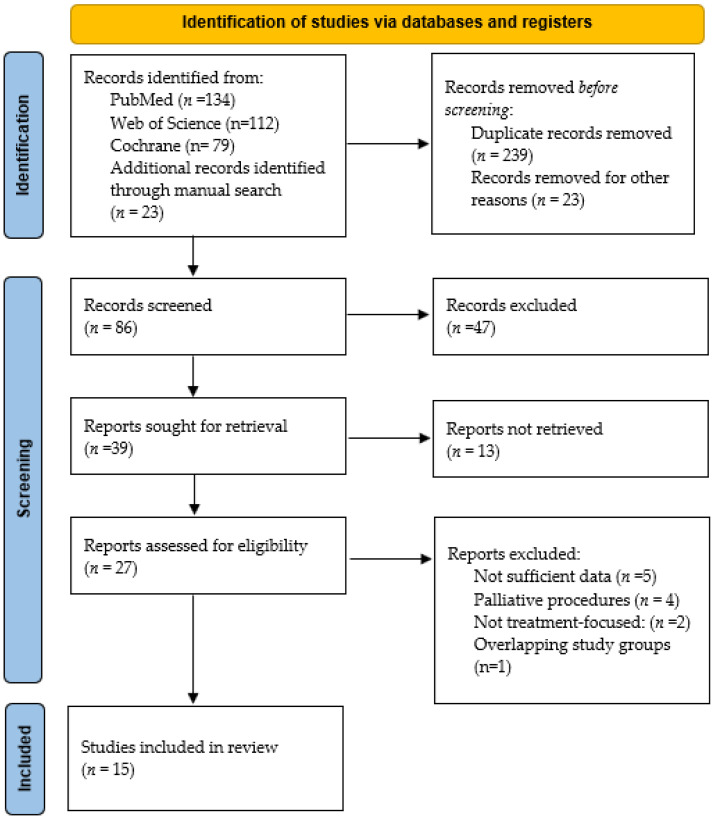
PRISMA flowchart for the studies included in the systematic review.

After removal of duplicates and application of the inclusion and exclusion criteria, 15 studies were included in the final analysis. The complete PRISMA flow diagram can be found in Figure 1.

### 2.4. Data Extraction

Data extraction was performed using a standardized, predesigned data collection form. Bibliographic variables included year of publication, first author, country of origin, and study design.

Extracted patient characteristics comprised sample size, age, type of complication (bowel obstruction or perforation), therapeutic intent (curative or palliative), tumor location, and the type of intervention performed in each comparative group.

The assessed outcomes included perioperative and 30-day mortality, overall postoperative morbidity, and major complications defined as Clavien–Dindo grade ≥ III. Additional surgical outcomes included surgical approach (open vs. laparoscopic), anastomotic leak rate, and stoma-related outcomes, including stoma creation and permanent stoma rates.

Long-term oncological outcomes included overall survival (OS), disease-free survival (DFS), and local recurrence rates.

For colonic stent placement, additional procedure-specific outcomes were extracted, including clinical success, procedure-related complications, and stent patency. Dichotomous outcomes were reported as proportions, survival outcomes as hazard ratios, when available, and continuous variables as reported in the original studies.

### 2.5. Quality Assessment

Methodological quality assessment was independently performed by two reviewers following study selection. Studies were first categorized according to study design as randomized controlled trials or observational studies.

Observational studies were evaluated using the Newcastle–Ottawa Scale (NOS), which assesses methodological quality across three domains: selection of study groups, comparability of cohorts, and ascertainment of outcomes. Studies with NOS scores ≥ 8 were considered high quality, scores of 6–7 were considered moderate quality, and scores < 6 were considered low quality. Randomized controlled trials were assessed using the revised Cochrane Risk of Bias tool (RoB 2), according to established methodological recommendations. The evaluation included assessment of the randomization process, deviations from intended interventions, missing outcome data, outcome measurement, and selection of reported results.

Quality assessment was performed independently by two reviewers, and disagreements were resolved through discussion and consensus. A third reviewer was consulted when necessary.

Detailed quality appraisal results are summarized in Table 2.

Observational studies included in the review were predominantly of moderate to high methodological quality, with NOS scores ranging from 6 to 9 points. The principal limitation identified among observational studies was reduced comparability between treatment groups, mainly related to baseline differences in patient severity, surgical risk, and treatment allocation. Among randomized controlled trials, the ESCO trial [19,20] demonstrated some concerns according to RoB 2 assessment, whereas the CReST trial [28] was classified as high risk of bias, primarily due to deviations from intended interventions and treatment crossover following stent failure.

Across the included studies, several potential sources of bias were consistently identified. In randomized trials, crossover from SEMS placement to emergency surgery due to stent failure represented the major methodological limitation. In addition, blinding was not feasible because of the nature of surgical and endoscopic interventions, introducing potential performance bias, particularly for subjective postoperative outcomes. Variability in operator expertise across multicenter studies may have further contributed to heterogeneity. Across both designs, misclassification of interventions, incomplete follow-up for long-term outcomes, and heterogeneity in definitions of outcomes further contribute to bias. Overall, the most important limitations are confounding by indication in observational studies and treatment crossover in RCTs, both of which are inherent to the emergency clinical context.

### 2.6. Data Analysis and Synthesis

Data extracted from the included studies were synthesized qualitatively and quantitatively, where appropriate. Descriptive analysis was initially performed to summarize study characteristics, including study design, patient demographics, tumor location and stage, therapeutic strategy, perioperative outcomes, and long-term oncologic results.

For comparative outcomes between acute resection (AR) and bridge to surgery using self-expandable metallic stents (BTS-SEMSs), pooled analyses were conducted using a random-effects model to account for expected clinical and methodological heterogeneity among studies. Dichotomous outcomes, including postoperative mortality, morbidity, laparoscopic surgery, and primary anastomosis rates, were analyzed using risk ratios (RRs) with corresponding 95% confidence intervals (CIs). A *p*-value < 0.05 was considered statistically significant. Long-term oncologic outcomes, including overall survival (OS) and disease-free survival (DFS), were synthesized by qualitative comparison, due to heterogenous reporting.

Statistical heterogeneity between studies was assessed using the Higgins I^2^ statistic and Cochran’s Q test. Heterogeneity was interpreted as low (I^2^ < 25%), moderate (25–50%), substantial (50–75%), or considerable (>75%). Given the variability in study populations, tumor stages, treatment intent, and timing of surgery after decompression, a random-effects approach was considered more appropriate than a fixed-effects model. Publication bias was evaluated through funnel plot inspection and formally assessed using Egger’s regression test and Begg’s rank correlation test. Sensitivity analyses were performed when appropriate, to evaluate the robustness of pooled estimates and the influence of individual studies on overall results.

Data were analyzed using Microsoft Excel and Med Calc^®^ Statistical Soft-ware (version 22.006, Med Calc Software Ltd., Ostend, Belgium). The research was conducted according to PRISMA checklist, as detailed in Supplementary File S1 [31].

## 3. Results

A total of 15 studies [15,16,17,18,19,20,21,22,23,24,25,26,27,28,29,30] published between 2015 and 2025, were included in this systematic review. The majority were retrospective in design and collectively included 6999 patients, of whom 4944 underwent acute resection (AR) and 1739 were treated by colonic stenting as bridge to surgery, while the remaining 311 underwent other BTS modalities. In three studies the two techniques were compared with decompression stoma [15,16,17], while Endo et al. [25] compared AR and colonic stenting with transanal decompression tube. De Roos et al. [24] compared postoperative outcomes based on the time frame from the acute presentation: acute resection, early resection (<4 weeks), and delayed resection (>4 weeks), including both stenting and stoma in the last two groups.

The mean age of the patients included in the reviewed studies was 70.6 (64.5–74.3) years old for the AR group and 69.8 (63.9–75.1) for the BTS-stent subgroup. A recurring pattern was that patients undergoing acute resection (AR) tended to be slightly older than those managed with bridge-to-surgery (BTS) strategies, including stenting or stoma formation [16,17,18,22,23]. In several studies, this difference was modest but noticeable [28,30], suggesting that older and potentially more fragile patients were more likely to undergo emergency surgery. However, this distinction was not uniformly reported across all cohorts, and in some studies the age distribution between groups was comparable [15,19,24].

Regarding sex distribution, the proportion of males and females was relatively balanced across studies, with a slight predominance of male patients in most cohorts. Overall, the male ratio was of 52.3% in the AR group and 57.2% in the stenting group. While some individual studies showed either male or female predominance, particularly in smaller subgroups, there was no consistent pattern indicating a significant sex-based difference between treatment groups. The main characteristics of the included studies are summarized in Table 3.

In several studies, patients undergoing emergency surgery had a higher prevalence of adverse prognostic factors, including higher ASA scores, partially explaining the reduced use of minimally invasive techniques [15,16,17]. Variations were also observed in surgical techniques and the extent of resection, with procedures performed either via open or laparoscopic approaches, depending on patient stability and institutional expertise.

The outcomes assessed across the included studies encompassed both short-term perioperative results and long-term oncologic endpoints, although the extent and consistency of reporting varied. Early outcomes, typically evaluated within 30 days, included postoperative mortality and morbidity, complication rates (particularly those related to stent placement), and surgical parameters such as the rate of primary anastomosis and the use of laparoscopic approach. Several studies also examined procedure-specific outcomes, including lymph node harvest [17] and the likelihood of permanent stoma formation. In contrast, long-term outcomes focused primarily on oncologic effectiveness, most commonly overall survival (OS) and disease-free survival (DFS), with follow-up periods ranging from three to five years. While most studies reported both early and delayed outcomes, some were limited to short-term endpoints only [15,16,17], contributing to heterogeneity in outcome reporting and limiting direct comparability across studies.

### 3.1. Early Postoperative Outcomes

Conventional management of patients with complicated colon cancer includes primary tumor resection or the creation of a decompressive stoma followed by delayed definitive surgery. Surgical approaches varied between open and minimal invasive approach, with a rate of laparoscopy varying between 2.9% [25] and 100% [22].

Self-expanding metal stents (SEMSs) were used as a bridge-to-surgery strategy for malignant bowel obstruction, with a reported clinical success rate varying from 78.6% to 97.3%, with a weighted mean of 89.1%. SEMS-related complications were infrequent overall, with pooled perforation rates estimated at approximately 5.6%, ranging from 0% to 15.5% across studies. Most contemporary series reported perforation rates below 5%, reflecting improved technical expertise and patient selection. Other adverse events, including stent migration, were rare (<3%). Although uncommon, perforation remains the most clinically significant complication due to its potential impact on both short- and long-term oncologic outcomes, particularly correlated with tumoral stage T4 in several studies [27,29] (Table 4).

#### 3.1.1. Laparoscopic Approach and Per-Primam Anastomosis

Colonic stenting as a bridge-to-surgery (BTS) strategy was associated with a significantly higher rate of laparoscopic surgery and primary anastomosis compared with acute resection (AR) in most studies. Among the 15 included studies, 14 reported data on laparoscopic surgery and were included in this analysis [15,16,18,19,20,21,22,24,25,26,27,29,30]. The rate of laparoscopy was consistently higher in the BTS-stent group, reflecting the conversion of an emergency presentation into a semi-elective setting following colonic decompression, which allows improved patient optimization, better intraoperative conditions, and more structured surgical planning. However, three studies [19,21,26] reported comparable laparoscopic rates between groups, likely reflecting high institutional expertise in minimally invasive colorectal surgery and more selective use of emergency laparotomy in both arms.

**Figure 2:**
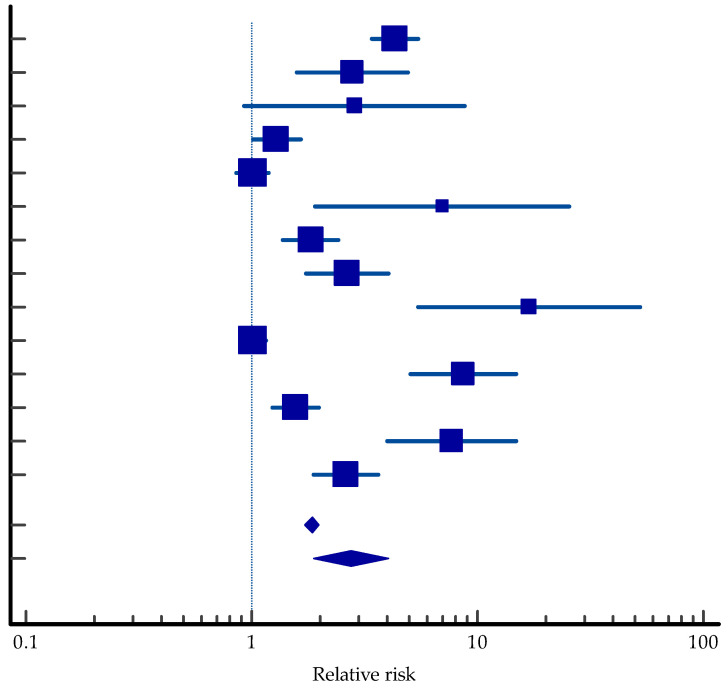
Relative risk (RR) associated with laparoscopic surgery in acute resection group vs. colonic stenting as bridge to surgery, followed by curative resection; Tanis et al., 2015 [15]; Amelung et al., 2016 [16]; Öistämö et al., 2016 [17]; Arezzo et al., 2017 [20]; Kim et al., 2017 [21]; Ji et al., 2017 [22]; Lara-Romero et al., 2019 [23]; de Roos et al., 2021 [24]; Endo et al., 2021 [25]; Katsuki et al., 2021 [26]; Hidalgo-Pujol et al., 2022 [27]; CReST Collaborative Group, 2022 [28]; Lin et al., 2024 [29]; Chen et al., 2025 [30]; total (fixed effects); total (random effects).

A random-effects meta-analysis demonstrated a significantly higher pooled laparoscopic rate in the BTS-stent group compared with AR (57.1%, 95% CI 51.2–62.7 vs. 13.6%, 95% CI 10.8–16.9), corresponding to a risk ratio (RR) of 2.76 (95% CI 1.89–4.03; *p* < 0.001) for laparoscopic surgery (Figure 2).

Substantial heterogeneity was observed in both groups (I^2^ = 95.3%), reflecting variability in tumor stage distribution, surgical timing, and institutional practice patterns. Egger’s test indicated potential funnel plot asymmetry (*p* = 0.036), whereas Begg’s test was not significant (*p* = 0.87). However, given the substantial heterogeneity (I^2^ = 95%), these results should be interpreted with caution, as asymmetry may reflect between-study variability, rather than publication bias. Despite this heterogeneity, the direction of effect was consistent across studies.

**Figure 3:**
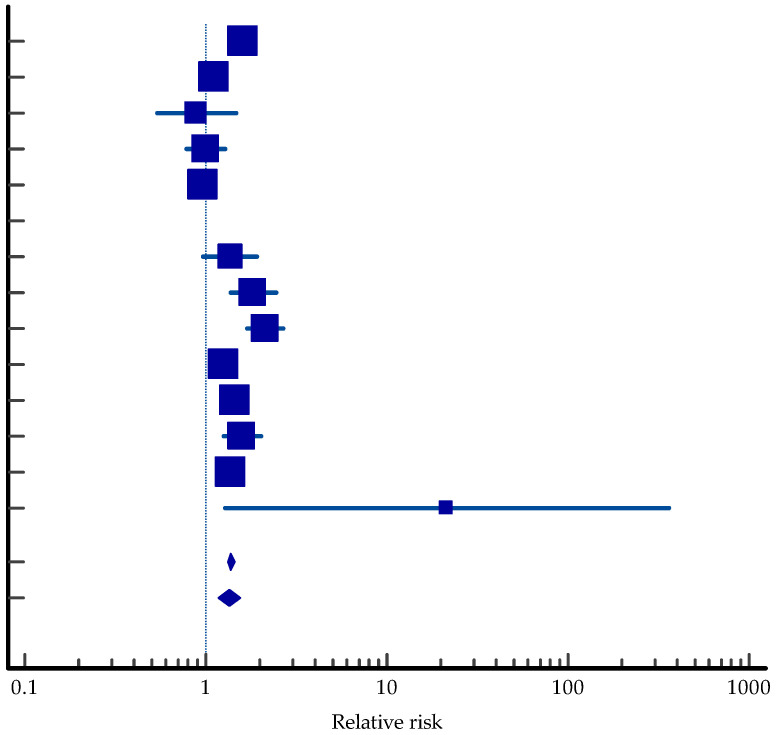
Relative risk (RR) for primary anastomosis in acute resection group vs. colonic stenting as bridge to surgery, followed by curative resection; Tanis et al., 2015 [15]; Amelung et al., 2016 [16]; Öistämö et al., 2016 [17]; Arezzo et al., 2017 [20]; Kim et al., 2017 [21]; Ji et al., 2017 [22]; Lara-Romero et al., 2019 [23]; de Roos et al., 2021 [24]; Endo et al., 2021 [25]; Katsuki et al., 2021 [26]; Hidalgo-Pujol et al., 2022 [27]; CReST Collaborative Group, 2022 [28]; Lin et al., 2024 [29]; Chen et al., 2025 [30]; total (fixed effects); total (random effects).

Across the included studies, bridge to surgery with self-expandable metallic stents (BTS-stent) was associated with a significantly higher rate of primary anastomosis compared with acute resection (AR), based on a random-effects meta-analysis (RR = 1.35, 95% CI 1.18–1.55, *p* < 0.001). The pooled stoma rate was 60.2% (95% CI 52.1–67.8) in the AR group and 27.4% (95% CI 21.6–34.1) in the BTS-stent group. This suggests that patients undergoing preoperative colonic decompression are more likely to undergo definitive surgery with restoration of bowel continuity, rather than requiring stoma formation (Figure 3).

However, the analysis demonstrated very high statistical heterogeneity (I^2^ = 92.3%, *p* < 0.0001), indicating substantial variability in effect estimates across studies and limiting the certainty of the pooled result. This heterogeneity is likely multifactorial, reflecting differences in patient populations (including variation in tumor location, disease stage, and inclusion of curative versus mixed-intent cohorts), differences in BTS strategies (stent-only approaches versus mixed-bridge techniques and variability in timing of elective surgery), and temporal changes in surgical practice such as increasing adoption of minimally invasive techniques and evolving thresholds for primary anastomosis. In addition, a small number of large studies contributed disproportionate statistical weight, while individual studies showed wide variability in effect sizes, ranging from no difference between groups to very large benefits in favor of BTS-stent. Despite this heterogeneity, there was no statistical evidence of publication bias according to Egger’s (*p* = 0.258) or Begg’s test (*p* = 0.807), although interpretation of these tests is limited in the presence of substantial between-study variability. Nevertheless, the consistency of the effect across studies supports BTS-stenting as a strategy that facilitates primary anastomosis and reduces the need for stoma formation in obstructive colorectal cancer. Thus, using BTS strategies involving colon stenting may improve the quality of life of colorectal patients in the early postoperative period [23,26].

#### 3.1.2. Early Postoperative Mortality and Morbidity

**Figure 4:**
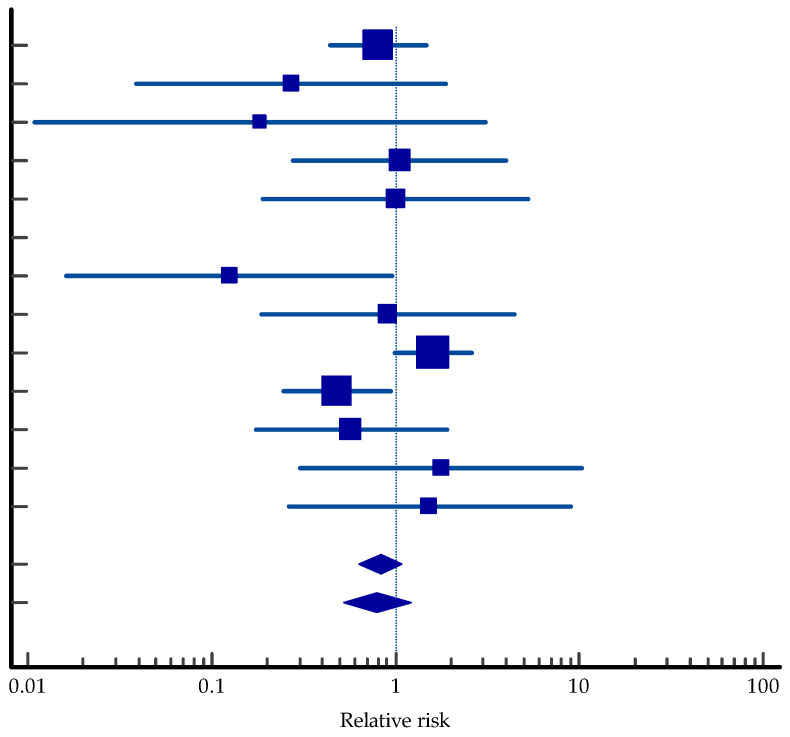
Relative risk for mortality in BTS-stent vs. AR group; Tanis et al., 2015 [15]; Amelung et al., 2016 [16]; Öistämö et al., 2016 [17]; Arezzo et al., 2017 [20]; Kim et al., 2017 [21]; Ji et al., 2017 [22]; de Roos et al., 2021 [24]; Endo et al., 2021 [25]; Katsuki et al., 2021 [26]; Hidalgo-Pujol et al., 2022 [27]; CReST Collaborative Group, 2022 [28]; Lin et al., 2024 [29]; Chen et al., 2025 [30]; total (fixed effects); total (random effects).

Postoperative morbidity varied widely for both groups, ranging from 8.9% to 59.5% in the patients that underwent AR, and 2.7–55.3% in the colonic stenting group. Across 13 included studies comparing bridge to surgery with self-expandable metallic stents (BTS-stent) versus acute resection (AR) for mortality outcomes, no statistically significant difference was observed between the two treatment strategies (Figure 4).

The pooled analysis using a random-effects model showed no statistically significant difference in short-term mortality between bridge to surgery with self-expandable metallic stents (BTS-stent) and acute resection (AR), with a risk ratio (RR) of 0.799 (95% CI 0.527–1.212, *p* = 0.291), indicating comparable perioperative mortality between the two strategies. Heterogeneity was low to moderate (I^2^ = 35.4%), suggesting relatively consistent effects across studies, and no evidence of publication bias was detected (Egger’s *p* = 0.174; Begg’s *p* = 0.493), supporting the robustness of the estimate. Overall, BTS-stent does not appear to increase short-term mortality, and can be considered a safe alternative to emergency resection in appropriately selected patients.

Postoperative morbidity showed more variability across studies, with several reporting lower complication rates in the BTS-stent group [15,22,23,24,27], while others, such as Kye et al. [18] and Katsuki et al. [26], found no significant differences. The pooled results suggested lower overall morbidity with BTS-stent (33.1% vs. 43.2% in AR), although interpretation is limited by substantial heterogeneity (I^2^ = 78%). This heterogeneity is largely attributable to inconsistencies in outcome definitions, with studies variably applying Clavien–Dindo criteria, composite morbidity endpoints, or broader administrative definitions, and some including stent-related complications while others did not. These methodological differences reduce comparability across studies, and should be considered when interpreting the observed trend toward reduced morbidity with BTS-stent.

In subgroup analysis, both Amelung and Tanis found that mortality was significantly higher (up to 30%) in frail patients, those >70 years, and those with more than two comorbidities and ASA ≥ 3, suggesting that colon stenting as BTS might improve the prognostics. Anastomotic leak rates were generally lower in BTS-stent groups [26,27,29,30], but statistical significance was demonstrated in only one study [27].

De Roos et al. [24] further evaluated timing of surgery after decompression in 168 patients. Ninety-day mortality did not differ significantly between emergency and early-staged surgery. However, late-staged resection (>4 weeks) was associated with significantly lower mortality compared with both emergency and early surgery (*p* = 0.029 and *p* = 0.011, respectively). Late surgery was also associated with reduced complication rates and improved long-term survival outcomes [30]. However, use of a stent followed by a long interval between placement and surgical resection (especially after neoadjuvant chemotherapy) can be associated with stent migration and delayed perforation in these vulnerable patients [15,16]. These findings may suggest that in patients that require longer preoperative preparation, including neoadjuvant therapy, decompression stoma may be a better option as BTS, compared to colonic stenting.

Thirty-day mortality ranged from 0% to 15.3% overall. Several studies demonstrated a trend toward lower mortality with BTS strategies [17,21,27], while others found no statistical differences between the two therapeutic strategies [18,19,22,23,26,29]. De Roos et al. [24] analyzed comparatively early postoperative outcomes in AR, early BTS, and delayed-BTS groups, and found that postponed surgery at 4 weeks or more after the acute obstructive event allows a better recovery, with significant lower mortality (1.8% vs. 12.2% and 15.3%) and fewer complications.

The pooled 30-day mortality was slightly lower in the BTS-stent group vs. the AR group (5.1% vs. 7.2%, *p* = 0.29); however, the differences were not statistically significant. Larger studies clustered closely around the pooled mortality rate, whereas smaller studies demonstrated greater variability, including both lower- (including zero-event) [18,22] and higher-mortality estimates [23]. Despite this variability, the direction of effect was consistent across studies, suggesting that BTS-stenting is associated with at least comparable, and potentially improved, short-term survival compared with immediate emergency resection.

Overall, early postoperative outcomes suggest that BTS—particularly with SEMS—provides important perioperative advantages, including higher technical success, increased use of minimally invasive surgery, and reduced stoma formation, while maintaining comparable mortality, and often lower morbidity. However, the risk of stent-related complications, especially perforation, remains a critical limitation and a potential determinant of both short- and long-term outcomes.

### 3.2. Long-Term Comparative Outcomes

Long-term oncologic outcomes, including overall survival (OS) and disease-free survival (DFS), were reported in the majority of included studies, with follow-up periods ranging from three to five years. Across these cohorts, outcomes were broadly comparable between patients undergoing acute resection (AR) and those managed with colonic stenting as a bridge to surgery. OS ranged from 42.6% to 77.7% in the AR group and 51.1% to 79.8% in the BTS-stent group, while DFS ranged from 45.7% [22] to 74.8% [25] and 46.8% [22] to 75.3% [23], respectively (Table 3). Most studies, including large multicenter analyses such as the CReST Collaborative Group [28], demonstrated no statistically significant differences in long-term oncologic outcomes between the two strategies [18,20,21,22,25,27]. However, some individual cohort studies reported variability, with improved survival outcomes in BTS groups in selected populations [23,26], likely reflecting selection bias and improved preoperative optimization, whereas other studies suggested potential disadvantages in specific subgroups treated by colonic stenting as BTS, particularly T4 tumors [20,29], possibly related to tumor biology or stent-related microperforation.

A key concern related to stenting is the risk of tumor dissemination following perforation, which has been hypothesized to negatively affect oncologic outcomes. Although perforation rates were relatively low, several studies emphasized that this complication—especially in advanced (T4) tumors—may contribute to worse recurrence patterns, including peritoneal spread [25,29].

Although most studies reported comparable OS and DFS between AR and BTS-stent groups, a formal pooled meta-analysis of survival outcomes was limited by inconsistent reporting of hazard ratios and time-to-event data. Therefore, long-term oncologic outcomes were synthesized qualitatively.

Notably, timing of surgery after decompression may influence outcomes, as delayed BTS was associated with improved survival in some series [24]. Overall, the balance of evidence supports oncologic equivalence between BTS-stenting and emergency resection, with long-term outcomes driven primarily by tumor stage and biology, rather than surgical strategy. In the study by Lara-Romero et al. [23], higher DFS for patients with stage III colonic cancer in the SBT-stent group (69.7% vs. 30%, *p* = 0.004) was partially explained by a higher number of lymph nodes harvested during laparoscopic resection on an elective basis in the stented group, compared to emergency surgical resection [23].

However, other studies draw attention on the potential oncological risk associated with SEMS, especially in cases complicated by perforation [21,29]. Inserting a SEMS can induce the spread of cancer cells, due to the mechanical stress on cancer tissue, especially in T4 tumors [28]. Lin et al. found a higher rate of peritoneal recurrence in T4 colon cancers that were treated by SEMS, while Arezzo et al. described a higher incidence of liver metastasis compared to the AR group [20,29].

Despite differences in perioperative management, long-term oncologic outcomes are largely equivalent, with prognosis primarily determined by tumor stage and underlying biology, rather than the timing or type of initial surgical intervention.

### 3.3. Comparison of Reported Outcomes in Observational Studies and RCTs

Subgroup analyses demonstrated broadly similar trends between observational studies and randomized controlled trials (RCTs), although the magnitude and certainty of effects differed across outcomes.

For laparoscopy, both study designs suggested that the intervention increased the likelihood of performing laparoscopic surgery. However, the effect estimate was substantially larger and statistically significant in observational studies (RR 3.08), whereas the association did not reach statistical significance in RCTs (RR 2.05, *p* = 0.057). This discrepancy may reflect residual confounding, selection bias, and differences in patient selection inherent to observational studies. In addition, heterogeneity was extremely high in both subgroups, indicating considerable variability among studies.

In contrast, the findings for primary anastomosis were highly consistent between observational studies and RCTs. Both subgroups demonstrated a statistically significant increase in the rate of primary anastomosis, with very similar pooled-effect sizes (RR 1.34 vs. 1.38). This concordance across study designs strengthens the evidence supporting a true beneficial effect of the intervention on bowel continuity restoration.

Regarding 30-day mortality, neither observational studies nor RCTs showed a statistically significant reduction in mortality. The pooled estimates were remarkably similar between study designs (RR 0.75 vs. 0.79), suggesting a consistent neutral effect on short-term mortality. Notably, heterogeneity was moderate in observational studies but absent in RCTs, indicating more stable mortality findings in randomized evidence.

The substantial heterogeneity observed for laparoscopy and primary anastomosis outcomes likely reflects differences in study populations, disease severity, emergency versus elective settings, surgeon expertise, institutional protocols, perioperative management strategies, and variations in the definition or implementation of the intervention across studies.

Overall, observational studies tended to demonstrate larger treatment effects, particularly for laparoscopy, whereas RCTs provided more conservative and methodologically robust estimates. The most reliable and consistent finding across both study designs was the increased likelihood of primary anastomosis without a corresponding reduction in 30-day mortality (Table 5).

## 4. Discussion

This systematic review evaluated the comparative effectiveness of acute resection (AR) versus self-expanding metal stent (SEMS) placement as a bridge-to-surgery (BTS) strategy in patients with obstructive colorectal cancer. Overall, BTS using SEMS is associated with improved short-term outcomes, including higher rates of minimally invasive surgery and primary anastomosis, as well as reduced stoma formation, while maintaining comparable short-term mortality and broadly equivalent long-term oncologic outcomes.

A consistent finding across the included studies is the increased use of minimally invasive surgery in BTS cohorts. This likely reflects the conversion of an emergency presentation into a semi-elective setting, allowing for colonic decompression and physiological optimization prior to definitive surgery. Consequently, patients in the BTS group are more frequently eligible for laparoscopic resection, which is associated with faster recovery and reduced postoperative morbidity. Several studies, including those by Tanis et al. [15] and Amelung et al. [16], demonstrated significantly higher laparoscopic resection rates following stent placement. Ji et al. [22] reported laparoscopic access in more than 90% of BTS cases compared with 12% in the AR group. In contrast, Öistämö et al. [17] observed no significant difference between groups, likely reflecting institutional expertise and selective use of laparoscopy in emergency settings. Overall, BTS consistently facilitates minimally invasive surgery, although the magnitude of benefit appears influenced by institutional experience and surgical-practice patterns.

BTS was also associated with higher rates of primary anastomosis and reduced stoma formation. This finding is clinically important, given the substantial impact of stoma creation on quality of life. Tanis et al. [15] reported significantly higher primary anastomosis rates in the BTS group (77.6% vs. 48.3%), a result corroborated by Amelung et al. [16] and Kim et al. [21]. The CReST Collaborative Group [28] similarly demonstrated a significantly reduced stoma rate in BTS patients, supporting the generalizability of this finding. These advantages likely reflect improved intraoperative conditions following decompression, as well as greater surgical confidence in performing primary anastomosis in a non-emergency setting [32,33]. Collectively, these findings indicate that BTS increases the likelihood of restorative surgery and reduces the need for stoma formation.

Short-term morbidity and mortality outcomes were heterogeneous across studies. Tanis et al. [15] and Amelung et al. [16] reported lower or comparable mortality in BTS groups, whereas Ji et al. [22] observed no perioperative deaths in either cohort. In contrast, Kye et al. [18] and Katsuki et al. [26] reported no significant differences in early mortality or complications. The large CReST trial [28] similarly demonstrated comparable short-term mortality between strategies. However, most reports demonstrated comparable perioperative mortality between BTS and AR strategies. This variability likely reflects differences in baseline patient characteristics, including ASA score, age distribution, and tumor burden at presentation [32,33]. Importantly, studies with higher-risk emergency cohorts tended to report worse outcomes in the AR group, suggesting confounding by indication. Overall, BTS does not appear to increase short-term mortality compared with emergency resection.

In malignant bowel obstruction, the rationale for a staged approach is physiologically sound. Colonic decompression may reduce bowel distension and improve tissue perfusion, thereby decreasing the risk of ischemia, perforation, and septic complications [34,35]. In addition, this approach allows physiological optimization in a patient population that is often elderly, frail, and metabolically compromised at presentation, enabling increased use of laparoscopic surgery [15,16,18,22,24,25,27,29,30] and higher rates of primary anastomosis [15,24,25,27,29]. Moreover, it may facilitate earlier initiation of systemic therapy, potentially improving tumor resectability and survival outcomes [36]. However, interpretation is limited by substantial baseline imbalances between groups, including older age (>70 years), ASA score > 3, and lower use of laparoscopy in emergency cohorts [15], which may confound direct comparisons [36]. Nevertheless, our findings appear broadly aligned with those of a recent meta-analysis encompassing 9493 patients with left-sided colon cancer, of whom 71.3% underwent emergency surgery and 9.6% were managed with a diverting stoma as BTS [34]. Emergency resection is associated with higher perioperative morbidity and less favorable short-term outcomes, although oncologic results may be comparable when technically successful. Differences in baseline characteristics—including age, comorbidities, and physiological status at presentation—may significantly influence outcomes and confound direct comparisons between treatment groups [37,38].

Metal stents were developed more than 20 years ago [39], and can be used as decompressive therapy for bowel obstruction, converting an emergency intervention into a semi-elective intervention. This minimally invasive approach represents one of the earliest forms of bridge-to-surgery strategies [40]. Performed using a flexible endoscope under fluoroscopic guidance, this approach fulfills the criteria of a bridging therapy, offering avoidance of laparotomy, shorter procedural time, feasibility under minimal sedation, and improved physiological optimization, including better control of comorbidities and nutritional status [41], with favorable short-term outcomes [42]. Initial randomized controlled trials comparing SEMS with emergency surgery were prematurely terminated, due to low stent-placement success rates [42,43], higher complication rates, and increased short-term morbidity, thereby raising early concerns regarding the safety of this strategy. However, these trials were conducted during the early adoption phase of SEMS, and likely reflect a significant learning-curve effect. In contrast, more recent evidence from multicenter studies and meta-analyses, including the CReST collaborative data, indicates that in experienced hands, SEMS can be performed safely, with acceptable complication rates and favorable perioperative outcomes. Technical success rates for SEMS placement have improved considerably over time, increasing from approximately 70% in early experiences in the 1990s to 84–94% in contemporary series, likely reflecting advances in technique, improved patient selection, and increased operator experience in high-volume centers [44,45]. In a meta-analysis of Cao et al., focused on the potential impact of SEMS-procedure complications on long-term outcomes, the authors found a significant survival benefit, with a success rate of SEMS placement ≥95% [46]. Overall, SEMS has transitioned from an experimental intervention to a standardized bridge-to-surgery strategy. Outcomes are strongly dependent on institutional experience and technical success.

A key question addressed in this review is whether the short-term advantages of BTS are achieved at the expense of oncologic safety. Overall, long-term outcomes, including overall survival (OS) and disease-free survival (DFS), were largely equivalent between BTS-stent and AR groups [15,16,17,18,19,20,22,23,24,25,26,27]. Nevertheless, some studies reported higher recurrence rates or worse survival associated with SEMS placement [21,28,29], particularly in cases complicated by perforation. Importantly, more recent population-based data have challenged this association, suggesting that earlier findings may reflect study selection and early procedural experience. These findings indicate that complications, rather than the stent itself, are the critical determinant of oncologic risk. Overall, stent-related perforation appears to be the key modifier of oncologic outcomes.

Stent placement and tumor manipulation may lead to tumor cell dissemination into the circulation, potentially increasing metastatic risk [47]. Several studies have raised concerns regarding worse oncologic outcomes following stent-related perforation [47,48,49,50,51]. Maruthachalam et al. [48] demonstrated increased cytokeratin 20 mRNA expression following stent insertion compared with diagnostic colonoscopy. Microperforations, which may be clinically silent, could also trigger a pro-tumoral inflammatory response [49,50,51]. Experimental studies in animal models have reported reduced survival and increased metastatic spread following stent placement [50,51]. Mechanical expansion of the tumor during deployment may facilitate tumor cell dissemination, particularly in locally advanced disease. This risk appears most relevant in T4 tumors, highlighting the importance of careful patient selection [50,51,52].

The timing of surgery after decompression may also influence outcomes. De Roos et al. [24] demonstrated that delayed resection (>4 weeks) was associated with lower morbidity and improved survival compared with early or emergency surgery. However, optimal timing remains uncertain and heterogeneous, across studies. Timing should therefore be individualized based on clinical recovery, patient optimization, and institutional practice.

Previous systematic reviews and meta-analyses have consistently shown that SEMS as a bridge-to-surgery strategy improves short-term outcomes, including higher primary anastomosis rates and reduced stoma formation, without increasing perioperative mortality [52,53,54,55,56]. Early meta-analyses, such as Zhang et al. [53], demonstrated that SEMS significantly improves primary anastomosis rates and reduces stoma formation without increasing peri-operative mortality, supporting its short-term surgical benefit in appropriately selected patients. More recent meta-analyses [46,54,55,56] have confirmed equivalent long-term oncologic outcomes between SEMS and emergency resection when procedures are performed in experienced centers. The meta-analysis by Cao et al. [46], including over 2500 patients, demonstrated comparable 3- and 5-year survival outcomes. Amelung et al. [54] similarly reported no significant differences in long-term survival, although sensitivity analyses suggested residual uncertainty in early randomized data. Contemporary evidence [55,56] further supports oncologic equivalence, emphasizing that outcomes are strongly dependent on technical success and complication rates, particularly stent-related perforation. Overall, BTS-stenting appears to improve perioperative outcomes, while maintaining oncologic safety in appropriately selected patients. However, careful patient selection and treatment in experienced centers remain essential [29,57,58,59]. SEMS may be most appropriate in patients who benefit from physiological stabilization before surgery [29]. Although there are limited data regarding the optimal timing, current studies support the concept that bridge-to-surgery strategies may be most effective when they truly convert an emergency presentation into an elective or semi-elective oncological procedure, rather than simply postponing surgery by a few days [58,59,60,61,62]. In contrast, immediate resection or decompressive stoma may be preferable in patients with contraindications to stenting or at high risk of perforation [63,64,65,66,67,68].

This systematic review provides an updated synthesis of contemporary evidence comparing acute resection with colonic stenting as a bridge-to-surgery strategy in obstructive colorectal cancer. Unlike earlier meta-analyses, which focused mainly on perioperative outcomes and early stent experience, it integrates modern surgical and endoscopic practice, while evaluating both short-term outcomes and long-term oncologic safety. Our findings refine current evidence by highlighting oncologic equivalence between strategies when applied in appropriately selected patients in experienced centers, while emphasizing the central role of technical success and stent-related complications in determining outcomes.

This review has several limitations. The majority of included studies were retrospective, and therefore subject to confounding by indication. Heterogeneity in patient populations, surgical techniques, and outcome definitions further limits comparability. The inclusion of palliative patients may have influenced outcome interpretation, due to their distinct clinical trajectory. In randomized controlled trials, crossover and protocol deviations may also have attenuated observed differences between groups. Finally, incomplete follow-up and variability in reporting of outcomes may affect the robustness of long-term conclusions.

Future research should focus on well-designed prospective studies with standardized outcome definitions and stratification by tumor stage, particularly T4 disease. Further investigation into the optimal timing of surgery after decompression and the long-term impact of stent-related complications is also needed. Separate analyses should be encouraged for right- versus left-sided obstruction, curative versus palliative settings, and localized versus diastatic perforation [69]. For SEMS, further work is also needed to clarify the oncological implications of stent-related microperforation and to evaluate whether improvements in device design and biomaterials can reduce procedure-related risk without compromising technical success [70].

## 5. Conclusions

Colonic stenting as a bridge-to-surgery strategy offers important short-term advantages, including reduced stoma formation, increased use of minimally invasive surgery, and improved postoperative recovery, while maintaining long-term oncologic outcomes comparable to emergency resection. Overall survival appears to be driven primarily by tumor biology, rather than surgical strategy; however, outcomes are influenced by careful patient selection, procedural expertise, and avoidance of complications, particularly perforation. This systematic review supports oncologic equivalence between strategies in appropriately selected patients based on contemporary evidence and highlights the central role of technical success in optimizing outcomes. Nevertheless, some uncertainty remains regarding specific high-risk subgroups, particularly patients with T4 tumors and those experiencing stent-related perforation, where potential oncologic risks may offset the short-term benefits of the bridge-to-surgery approach.

Future research should focus on prospective, ideally multicenter, studies, with standardized outcome definitions, stratification by tumor stage—especially T4 disease—and more precise evaluation of optimal timing of surgery following decompression. Such studies are needed to better define high-risk subgroups and further refine treatment selection strategies.

## Figures and Tables

**Table 1:** Eligibility criteria for the studies included in the systematic review.

Component	Definition
Population	Adult patients (≥18 years) with primary colon cancer presenting with bowel obstruction
Intervention	Self-expanding metallic stent (SEMS) used as a bridge-to-surgery strategy
Comparison	Emergency surgical resection for malignant colon obstruction
Outcomes (Primary)	30-day mortality, severe postoperative morbidity (Clavien–Dindo ≥ III), stoma-related outcomes (primary anastomosis rate, permanent stoma rate)
Outcomes (Secondary)	Overall survival (OS), disease-free survival (DFS), recurrence

**Table 2:** Quality appraisal of the studies included in the review.

Author & Year	Country	Study Design	Quality Appraisal Tool	Score
Tanis et al. (2015) [15]	Netherlands	Prospective national registry (Stent-In II trial)	NOS	🟩 9/9
Amelung et al. (2016) [16]	Netherlands	Population-based comparative	NOS	🟩 9/9
Öistämö et al. (2016) [17]	Sweden	Retrospective cohort	NOS	🟨 7/9
Kye et al. (2016) [18]	South Korea	Multicenter retrospective	NOS	🟨 6/9
Arezzo et al. (2017)/2019 [19,20]	Italy/Spain	Multicenter RCT (ESCO trial)	RoB 2	🟨 Some concerns
Kim et al. (2017) [21]	South Korea	Multicenter comparative	NOS	🟨 7/9
Ji et al., 2017 [22]	South Korea	Multicenter retrospective comparative cohort	NOS	🟨 7/9
Lara-Romero et al. (2019) [23]	Spain	Bicentric retrospective	NOS	🟨 7/9
de Roos et al. (2021) [24]	Netherlands	Retrospective cohort (prospective database)	NOS	🟩 8/9
Endo et al. (2021) [25]	Japan	Multicenter observational	NOS	🟩 9/9
Katsuki et al. (2021) [26]	Japan	Retrospective cohort	NOS	🟩 9/9
Hidalgo-Pujol et al. (2022) [27]	Spain	Multicenter observational	NOS	🟩 8/9
CReST Collaborative Group (2022) [28]	UK	Randomized clinical trial	RoB 2	🟥 High risk
Lin et al. (2024) [29]	Singapore	Retrospective cohort	NOS	🟨 6/9
Chen et al. (2025) [30]	China	Retrospective comparative	NOS	🟨 7/9

Footnote: NOS—Newcastle–Ottawa Scale; RoB 2—Cochrane Risk of Bias tool. 🟩 High methodological quality/low risk of bias; 🟨 moderate quality/some concerns; 🟥 high risk of bias.

**Table 3:** General characteristics of the studies included in the systematic review.

Author & Year	Intervention(s) Compared	*N*	Age (Mean, yrs)	M:F Ratio	Tumor Location	Tumor Stage	Therapeutic Intention	Time of Surgery After BTS Procedure	Reported Outcomes
Tanis et al., 2015 [15]	AR vs. BTS (SEMS; stoma)	1816 (1485 AR; 196 BTS-stent; 135 stoma)	70 (AR); 71 (stent); 68 (stoma)	805:680; 119:77; 76:59	Left-sided colon	II–IV	Mixed	Not reported	30-day morbidity/mortality; SEMS complications; primary anastomosis; laparoscopic use
Amelung et al., 2016 [16]	AR vs. BTS (SEMS; stoma)	1860 (1774 AR; 42 BTS-stent; 44 stoma)	71.4; 69.9; 64.9	858:916; 20:24; 24:18	Right/transverse colon	II–IV	Mixed	28.1 vs. 109.9 days	30-day morbidity/mortality; anastomosis rate
Öistämö et al., 2016 [17]	AR vs. BTS (SEMS; stoma)	100 (57 AR; 20 BTS-stent; 23 stoma)	74 ± 12; 71 ± 10; 67 ± 12	31:26; 7:13; 13:10	Left-sided colon	II–III	Curative	Not reported	30-day outcomes; lymph node harvest; permanent stoma rate
Kye et al., 2016 [18]	AR vs. BTS (SEMS)	74 (49 AR; 25 BTS-stent)	70.8 ± 11.6; 69.4 ± 9.9	21:28; 14:11	Right-sided colon	II–III	Curative	~14 days	OS; DFS
Arezzo et al., 2017/2020 [19,20]	AR vs. BTS (stent)	115 (59 AR; 56 BTS-stent)	71; 72	32:27; 28:28	Left-sided colon	Not reported	Curative	~5 days	OS; DFS (3 years)
Kim et al., 2017 [21]	AR vs. BTS (stent)	168 (56 AR; 112 BTS-stent)	64.5 ± 13.5; 63.9 ± 12.5	30:26; 70:52	Left-sided colon	II–III	Curative	Not reported	OS; DFS (5 years)
Ji et al., 2017 [22]	AR vs. BTS (stent)	39 (15 AR; 14 BTS-stent)	66.9 ± 12.4; 61.5 ± 14.4	11:14; 4:10	Right-sided colon	II–IV	Curative	Variable (6.9 days early; delayed cases)	OS; DFS
Lara-Romero et al., 2019 [23]	AR vs. BTS (stent)	137 (66 AR; 71 BTS-stent)	73.5; 69	30:36; 40:29	Left-sided colon	I–III	Curative	Not reported	OS; DFS (5 years)
de Roos et al., 2021 [24]	Emergency resection vs. delayed surgery after decompression	168 (74 AR; 34 early BTS; 56 delayed BTS)	65.1 ± 12.9; 69.1 ± 10.7; 68.1 ± 11.9	34:40; 23:11; 35:21	Left-sided colon	II–IV	Curative	<4 weeks (early); >4 weeks (delayed)	OS; DFS
Endo et al., 2021 [25]	AR vs. BTS (stent vs. TAD tube)	301 (103 AR; 113 BTS-stent; 85 tube)	67; 69; 69	64:39; 69:44; 43:42	Left-sided colon	II–III	Curative	10–17 days	DFS (3 years)
Katsuki et al., 2021 [26]	AR vs. BTS (stent)	996 (498 AR; 498 BTS-stent)	72; 73	271:227; 272:226	Left-sided colon	II–III	Curative	~15 days	OS (5 years)
Hidalgo-Pujol et al., 2022 [27]	AR vs. BTS (stent)	564 (320 AR; 244 BTS-stent)	74.3; 75.1	191:129; 152:92	Left-sided colon	I–III	Curative	Not reported	OS; DFS (3 years)
CReST Collaborative Group, 2022 [28]	Stenting + elective surgery vs. decompression ± resection	245 (123 AR; 122 BTS-stent)	69.9 ± 12.2; 69.1 ± 11.2	72:51; 77:45	Transverse & left colon	II–IV	Mixed	1–4 weeks	OS; DFS (3 years)
Lin et al., 2024 [29]	AR vs. BTS (stent)	227 (165 AR; 62 BTS-stent)	66.9 ± 12.4; 68.5 ± 12.8	95:70; 37:25	Left-sided colon	II–III	Curative	10–14 days	OS; DFS (5 years)
Chen et al., 2025 [30]	AR vs. BTS (stent)	189 (100 AR; 98 BTS-stent)	66; 65	45:44; 56:44	Left-sided colon	II–III	Curative	Not reported	OS; DFS (5 years)

Footnote: AR—anterior resection; BTS—bridge to surgery; SEMS—Self-Expandable Metallic Stent; TAD—transanal decompression tube; OS—overall survival; DFS—disease-free survival. The majority of cohorts involved patients with obstructive colorectal cancer stage II–III, treated with curative intent; however, several studies also included mixed populations of stage II–IV, who underwent either curative or palliative surgery, which may be a potential cause of bias in reporting long-term postoperative outcomes [15,16,28]. Tumor location was predominantly left-sided, although some studies included right-sided and transverse colon tumors or mixed anatomical distributions [16,18,22,28]. Differences in baseline characteristics, including age, comorbidity burden, and physiological status at presentation, were inconsistently reported, but likely influenced treatment allocation [15,16,17,24].

**Table 4:** Comparative early and delayed outcomes for acute resection (AR) versus colonic stenting as bridge to surgery (BTS-stent) in obstructive colonic cancer.

Study	Clinical Success	SEMS Complications	30-Day Mortality	Morbidity	Laparoscopy/Primary Anastomosis	OS	DFS	Main Findings	Study Limitations
Tanis et al., 2015 [15]	No info	Perforation 10 (5.1%)	AR: 6.9%; BTS-stent: 5.6%; BTS-stoma: 3.7%	AR: 42.8%; BTS-stent: 31.3%; BTS-stoma: 28.2%	AR: 9.2%; BTS-stent: 39.5%; BTS-stoma: 25.2%	AR: 48.3%; BTS-stent: 77.6%; BTS-stoma: 72.2%	No info	BTS associated with higher laparoscopy and primary anastomosis; similar mortality/morbidity	Limited oncologic follow-up; incomplete survival data; heterogeneity in BTS group
Amelung et al., 2016 [16]	No info	No info	AR: 8.8%; BTS-stent: 2.4%; BTS-stoma: 2.4%	AR: 39.6%; BTS-stent: 27.3%; BTS-stoma: 31.7%	AR: 8.5%; BTS-stent: 22.7%; BTS-stoma: 9.5%	AR: 85.6%; BTS-stent: 95.5%; BTS-stoma: 90.5%	No info	BTS associated with higher laparoscopy and anastomosis; lower mortality	Registry-based; very small BTS subgroups; confounding by indication
Öistämö et al., 2016 [17]	No info	No info	AR: 12%; BTS-stent: 0%; BTS-stoma: 0%	AR: 22.8%; BTS-stent: 23%; BTS-stoma: 20%	No info	AR: 56%; BTS-stent: 50%; BTS-stoma: 94%	No info	BTS associated with lower mortality; similar complications	Small cohort; treatment heterogeneity
Kye et al., 2016 [18]	No info	No info	0% (both groups)	AR: 19.1%; BTS-stent: 24%	AR: 32%; BTS-stent: 60%	AR: 86.5 ± 6.9 months; BTS-stent: 115.7 ± 7.6 months	AR: 75.9 ± 6.3 months; BTS-stent: 97.8 ± 11.1 months	Comparable outcomes; BTS increases laparoscopy	Single-center; small sample size
Arezzo et al., 2017/2020 [19,20]	78.6%	Perforation 14.2%; (8.9%)	AR: 7.1%; BTS-stent: 5.1%	AR: 57.6%; BTS-stent: 51.8%	AR: 61%; BTS-stent: 77.8%	AR: 69.3%; BTS-stent: 70.1%	Similar at 36 months; liver mets: 6.8% vs. 12.5%	Meta-analysis heterogeneity; variable stent expertise	Limited staging information; observational design
Kim et al., 2017 [21]	92%	Perforation 8%	AR: 3.6%; BTS-stent: 0.9%	AR: 8.9%; BTS-stent: 2.7%	AR: 53.6%; BTS-stent: 58.9%	AR: 77.7%; BTS-stent: 79.7%	AR: 73.1%; BTS-stent: 69.5%	Favorable perioperative outcomes in BTS	Single-country cohort; selection bias
Ji et al., 2017 [22]	87.5%	0%	0% both groups	AR: 40%; BTS-stent: 7.1%	AR: 12%; BTS-stent: 93%	AR: 46.1%; BTS-stent: 51.1%	AR: 45.7%; BTS-stent: 46.8%	BTS improves perioperative outcomes	Retrospective; imbalance in groups
Lara-Romero et al., 2019 [23]	84.5%	Perforation 15.5%	AR: 7.6%; BTS-stent: 7%	AR: 28.8%; BTS-stent: 15.5%	No info	AR: 42.6%; BTS-stent: 57.7%	AR: 59.8%; BTS-stent: 75.3%	BTS reduces stoma; improved DFS in stage III	Non-randomized; staging imbalance
de Roos et al., 2021 [24]	No info	Perforation 7.5%	AR: 12.2%; early BTS: 15.3%; delayed BTS: 1.8%	AR: 59.5%; early BTS: 55.3%; delayed BTS: 35.7%	AR: 25.7%; early BTS: 60.6%; delayed BTS: 76.8%	AR: 51.4%; early BTS: 52.6%; delayed BTS: 75%	AR: 74.3%; early BTS: 63.2%; delayed BTS: 82.11%	Delayed BTS superior short- and long-term	Observational design; timing heterogeneity
Endo et al., 2021 [25]	97.3% (BTS-stent); 85.9% (BTS-tube)	2.7% (BTS-stent); 8.3% (BTS-tube)	AR: 2.9%; BTS-stent: 49.6%; BTS-tube: 24.7%	AR: 34.9%; BTS-stent: 20.3%; BTS-tube: 29.4%	AR: 43.7%; BTS-stent: 92.9%; BTS-tube: 50.6%	AR: 74.8%; BTS-stent: 69%	No info	BTS-stent improves perioperative outcomes	Mixed BTS modalities
Katsuki et al., 2021 [26]	No info	No info	AR: 5%; BTS-stent: 8%	AR: 22%; BTS-stent: 23%	AR: 52%; BTS-stent: 51%	AR: 71%; BTS-stent: 90%	No info	Lower stoma rate but worse OS in BTS	Retrospective; confounding
Hidalgo-Pujol et al., 2022 [27]	No info	7.8% (perforation 4.1%)	AR: 9.4%; BTS-stent: 4.5%	AR: 60%; BTS-stent: 41.5%	AR: 4.38%; BTS-stent: 37.7%	AR: 70.6%; BTS-stent: 79.8%	AR: 67.4%; BTS-stent: 70.4%	No long-term differences	Missing procedural timing data
CReST Collaborative Group, 2022 [28]	82.4%	Perforation 3.3%	AR: 5.6%; BTS-stent: 3.6%	AR: 37.7%; BTS-stent: 32.5%	AR: 42.7%; BTS-stent: 67.3%	AR: 60%; BTS-stent: 66.3%	AR: 66.4%; BTS-stent: 57.2%	BTS reduces stoma; similar oncologic outcomes	Moderate follow-up duration
Lin et al., 2024 [29]	91.9%	Perforation 1.62%	AR: 1.8%; BTS-stent: 3.2%	AR: 31%; BTS-stent: 21%	AR: 6.1%; BTS-stent: 46.8%	AR: 49.7%; BTS-stent: 58.1%	AR: 63.6%; BTS-stent: 54.8%	BTS may ↑ peritoneal recurrence in T4	Retrospective; stage-specific bias
Chen et al., 2025 [30]	90.8%	Perforation 3%; migration 3%	No info	AR: 2.9%; BTS-stent: 3%	AR: 29%; BTS-stent: 76%	AR: 0%; BTS-stent: 10%	Similar OS and DFS	BTS-stent is associated with ↑laparoscopy; ↓stoma rate; comparable long-term outcomes with AR	Early follow-up; limited long-term data

Footnote: AR—anterior resection; BTS—bridge to surgery; SEMS—self-expandable metallic stent; OS—overall survival; DFS—disease-free survival.

**Table 5:** Subgroup analysis for postoperative outcomes—observational studies vs. RCTs.

Outcome	Observational Studies (Random-Effects)	RCTs (Random-Effects)	Comparison/Interpretation
Laparoscopy	RR 3.08 (95% CI 1.94–4.90), *p* < 0.001, I^2^ = 95.1%	RR 2.05 (95% CI 0.98–4.30), *p* = 0.057, I^2^ = 96.5%	Both favored the intervention, but observational studies showed a larger and statistically significant effect. RCTs showed only borderline significance.
Primary anastomosis	RR 1.34 (95% CI 1.15–1.57), *p* < 0.001, I^2^ = 92.4%	RR 1.38 (95% CI 1.05–1.83), *p* = 0.021, I^2^ = 84.5%	Highly consistent findings across study designs, supporting a robust beneficial effect of the intervention.
30-day mortality	RR 0.75 (95% CI 0.40–1.42), *p* = 0.379, I^2^ = 51.2%	RR 0.79 (95% CI 0.48–1.31), *p* = 0.360, I^2^ = 0%	Neither subgroup demonstrated a mortality benefit. Mortality findings were more consistent in RCTs.
Heterogeneity pattern	High heterogeneity for surgical outcomes	High heterogeneity for surgical outcomes, low for mortality	Variability likely reflects differences in patient selection, surgical expertise, and perioperative management.
Overall interpretation	Larger estimated effects	More conservative estimates	Observational studies may overestimate benefit due to confounding and selection bias, whereas RCTs provide more robust causal evidence.

## Data Availability

No new data was created.

## Supplementary Materials

The following supporting information can be downloaded at: https://www.mdpi.com/article/10.3390/jcm15124416/s1, File S1: (PRISMA-ScR) Checklist [31].
